# Community Pharmacy Engagement in Diabetes Prevention: Key Informant Interviews with Pharmacy Executives

**DOI:** 10.5888/pcd17.200050

**Published:** 2020-08-20

**Authors:** Sara E. Roszak, Stefanie P. Ferreri

**Affiliations:** 1 Division of Practice Advancement and Clinical Education, UNC Eshelman School of Pharmacy, Chapel Hill, North Carolina

## Abstract

**Introduction:**

Even though evidence-based diabetes prevention interventions exist, more than 1 in 3 Americans have prediabetes; the use of pharmacies has been explored as a way to reach and care for this population. The objective of this study was to analyze factors that influence adoption of type 2 diabetes prevention programs by community pharmacies.

**Methods:**

We conducted 21 semistructured interviews in 2018 with decision makers from 11 independent pharmacies in 6 US states and the District of Columbia and from 10 chain pharmacies operating in 1 state, multiple states, and nationwide. We identified participants by using purposive sampling. We used qualitative methods to analyze data and conducted interviews until we reached saturation.

**Results:**

Multiple themes emerged: 1) initiation of services is more likely if initial financial support is received; 2) patient demand for services, actual or perceived, is paramount; 3) diabetes prevention services often fit within the existing operations of a pharmacy and allow maximum use of resources; 4) customer loyalty is a clearly articulated advantage against competition; and 5) engagement in diabetes prevention affirms an expanded role and the value of pharmacies to serve communities.

**Conclusion:**

Pharmacies are well situated to deliver diabetes prevention programs to communities. Although considerable opportunity exists for pharmacies to address diabetes prevention, more could be done to reduce barriers to their use.

**Summary**

**What is already known on this topic?**

Effective yet underused strategies exist to prevent or delay the onset of type 2 diabetes. Community pharmacies are accessible destinations for preventive and chronic care management, and some pharmacies offer diabetes prevention services, including diabetes testing and lifestyle-change interventions.

**What is added by this report?**

This study identified factors that influence whether community pharmacies adopt and implement diabetes testing and National Diabetes Prevention Program (DPP) lifestyle-change interventions.

**What are the implications for public health practice?**

Community pharmacies can offer diabetes testing and the National DPP lifestyle-change intervention, both of which support public health goals to increase awareness and action for people with prediabetes. Barriers to implementation of diabetes prevention programs should be addressed.

## Introduction

Diabetes imposes a societal and public health burden. More than 100 million Americans live with diabetes or prediabetes. Developing type 2 diabetes is a gradual but preventable process. In a 2019 study, nearly 1 in 5 adolescents aged 12 to 18 and 1 in 4 young adults aged 19 to 34 were living with prediabetes (1).

For people with prediabetes, reducing body weight and exercising can prevent or delay onset of type 2 diabetes. Studies demonstrated that such modifications resulted in a 30% to 60% reduction in diabetes incidence (2); these studies influenced the creation of the Centers for Disease Control and Prevention’s (CDC’s) National Diabetes Prevention Program (National DPP). This program offers evidence-based, cost effective interventions to prevent diabetes. The National DPP, a lifestyle-change intervention, is offered by programs that meet quality standards through its recognition program (Table 1).

**Table 1:** Diabetes Prevention Recognition Program (DPRP) Process, Adapted from Rx for the National Diabetes Prevention Program (DPP) Action Guide for Community Pharmacists^a^

Phase of Process	Steps in Process
Pre-application	• Read and understand the current DPRP standards. • Complete the organizational capacity assessment tool (strongly recommended). • Address any capacity gaps identified by the assessment. • Review other materials about the National DPP and DPRP on the National DPP’s website, Implement a Lifestyle Change Program (for Professionals).

Application submitted for recognition	• Complete the online DPRP application form.

Pending recognition	Meet the following requirements: • Submit a completed application. • Use a CDC-approved curriculum. • Offer a 12-month lifestyle-change program that includes a minimum of 16 weekly sessions in months 1 to 6 and 6 monthly sessions in months 7 to 12. Agree to: • Start the first session within 6 months of effective date. • Start at least 1 session every 12 months. • Submit required participant data to DPRP every 6 months.

Preliminary recognition	Meet the following requirements: • Submit required data every 6 months. • Start at least 1 session every 12 months. • Continue to meet the pending recognition requirements. • Submit a full 12 months of data on at least 1 completed group of participants. • Have a minimum of 5 participants who attended at least 3 sessions in months 1 to 6 and whose time from first session to last session was at least 9 months. • Provide evaluated data that show that at least 60% of participants attended at least 9 sessions in months 1 to 6 and at least 60% attended at least 3 sessions in months 7 to 12.

Full recognition	Meet the following requirements: • Submit required data every 6 months. • Start at least 1 session every 12 months. • Continue to meet the requirements for pending and preliminary recognition. • Body weight documentation: Participants must have had their body weight documented during at least 80% of sessions.^b^ • Physical activity documentation: Physical activity minutes must have been documented for participants during at least 60% of sessions.^b^ • Weight loss at 12 months: Average weight loss across all participants in 1-year–long program must be a minimum of 5% of starting body weight.^b^ • Participant eligibility: A minimum of 35% of all participants in 1-year–long program must be eligible on the basis of either a blood glucose test indicating prediabetes or a history of gestational diabetes. The rest must be eligible on the basis of a high score on the CDC Prediabetes Screening Test or the American Diabetes Association Type 2 Diabetes Risk Test.^b^^,^ c

a Centers for Disease Control and Prevention (3).

b Evaluation for these requirements based on all participants attending at least 3 sessions during months 1 to 6 and whose time from first session to last session is at least 9 months. At least 5 participants per submission who meet this criterion are required for evaluation.

c All Medicare Diabetes Prevention Program beneficiaries must have a blood glucose test for eligibility.

Several commercial and government payers provide coverage for the National DPP lifestyle-change intervention (Table 2). Expanding coverage is important to reach eligible participants and reduce financial burdens. Increases in the number of partnerships and access points are needed to prevent diabetes. Pharmacies are well-positioned to support this effort. CDC has promoted collaboration with pharmacies and pharmacists since its release of a guide supporting such action (3).

**Table 2:** Payer Coverage of the National Diabetes Prevention Program Lifestyle-Change Program as of 2018^a^

Type of Insurer	Name of Insurer
**Commercial insurers**	

Many commercial health plans provide some coverage for the National DPP lifestyle change program.	AmeriHealth Anthem Blue Cross Blue Shield Florida Blue Shield California Blue Cross Blue Shield Louisiana Cigna Denver Health Managed Care: Medicaid, Medicare, Public Employees Emblem Health: New York Government Employees Health Association Highmark Humana Kaiser: Colorado Kaiser: Georgia Louisiana Care: Medicaid MVP Health Care Medicare Advantage Priority Health: Michigan United Health Care (national, state, local, private, and public employees)

**US government**	

The Centers for Medicare and Medicaid Services issued a final rule that allows for coverage of the National DPP lifestyle change program on a pay-for-performance basis.	Medicare (April 2018) Medicaid

**State coverage**	

The National DPP is a covered benefit for more than 3.4 million public employees/dependents in 19 states. Demonstration projects ongoing in North Dakota, Pennsylvania, and Utah.	California Colorado Connecticut (Department of Transportation ) Delaware Georgia (Kaiser Permanente) Indiana Kentucky Louisiana Maine Maryland (partial payment) Minnesota New Hampshire New York Oregon (educators) Rhode Island Tennessee Texas Vermont Washington

a From Albright (4).

The current transformative health care landscape provides an opportunity to look beyond traditional models of care to increase access, reduce costs, and improve health outcomes. Despite mounting evidence on the value of pharmacists as patient care providers (5), legal, policy, and reimbursement frameworks have not kept pace nor adequately recognized pharmacists as providers.

Our study considers community pharmacies as a health care destination for diabetes prevention programs. Few studies have explored how pharmacies can affect diabetes prevention, and no study has considered factors that influence the decision making of pharmacy executives. The objective of our study was to identify factors that enable or hinder pharmacy adoption of diabetes prevention programs.

## Methods

We gathered data through semistructured interviews with a purposive sample of key informants that discussed their company’s engagement, or lack thereof, in diabetes prevention programs. We conducted interviews from May through August 2018 until we reached saturation of information and themes. To recruit key informants from pharmacies engaged in the National DPP lifestyle-change intervention, we reviewed CDC’s publicly available registry of 1,693 participating organizations on March 30, 2018. The registry listed 26 independent and 5 chain pharmacy corporate entities, and we contacted executives at these pharmacies. To identify the appropriate executives, the interviewer (S.E.R.) had access to chain pharmacy executives through personal connections and her employer, and she received a list of independent pharmacy contacts through the National Community Pharmacy Association. We sought a convenience sample of independent pharmacies not engaged in such programs through referrals from state pharmacy associations and pharmacy professors. For chain pharmacies, the interviewer contacted executives by using a list of the top 25 pharmacies by prescription share. This qualitative research study was deemed exempt by the institutional review board at the University of North Carolina, Chapel Hill.

We conducted interviews with 22 key informants, individuals who had the decision-making authority to determine whether their employer could adopt new patient care services. We excluded information from 1 key informant because we realized after the interview was complete that this person did not meet our eligibility criterion of having the appropriate decision-making authority. The 21 participants represented 11 independent and 10 chain pharmacies. We analyzed data from the 21 interviews by using MAXQDA (VERBI GmbH) to write memoranda, develop codes, and identify themes. We used well-documented qualitative data analysis techniques (6,7) which led to 5 equally weighted key themes. A second coder, an undergraduate student, reviewed all transcripts, and an intercoder reliability score of 83.3%, above the 80% benchmark, was reached. 

## Results

The 11 independent pharmacy executives were affiliated with pharmacies in Illinois, North Carolina, Pennsylvania, Tennessee, Texas, Utah, and the District of Columbia. The 10 chain pharmacy executives represented 3 traditional drug stores, 5 grocery stores with pharmacies, and 2 mass merchants with pharmacies. Half of the chain pharmacy participants worked for companies with a national presence, operating more than 1,000 stores. The other half worked for companies with a regional presence, including primarily companies operating 200 or more stores; one had fewer than 20 stores. Nearly all independent pharmacy participants had been with their company for up to 15 years. Conversely, more than half of chain pharmacy participants had been with their company for 16 years or more, with 4 chain pharmacy participants reporting 26 to 30 years.

More than half of companies interviewed delivered both diabetes testing and the National DPP lifestyle-change intervention. Five key themes emerged as factors that enable or hinder pharmacy adoption of diabetes prevention programs (Table 3).

**Table 3:** Major Themes and Findings on Community Pharmacy Engagement in Diabetes Prevention From Key Informant Interviews With Pharmacy Executives, 2018

Theme	Explanation of Theme	Finding	Representative Quote
Financial feasibility	Initiation of services is more likely if initial financial support is received and likely to result in a sustainable business model.	Financial feasibility and sustainable reimbursement models are critical for adoption of diabetes prevention programs, with grant funding a catalyst most commonly used by independent pharmacies and grocery stores with pharmacies.	An independent pharmacy participant said, “[W]e obviously are testing the waters and figuring things out in this beta version. And hopefully we’ll have all the kinks ironed out for our second go, which would be when we are Medicare-payment eligible.”

Consumer participation	Consumer buy-in and demand for services, actual or perceived; initiation and retention in diabetes prevention services is paramount.	Inadequate consumer participation in diabetes prevention programs is problematic, but pharmacies are committed to solving this issue.	An independent pharmacy participant said, “A lot of people don’t want to participate, they don’t want to take the time.”

Operational fit	Diabetes prevention services fit within the existing operational structure of a pharmacy and allow the pharmacy to maximize its personnel and resources.	Operational fit is important, and appropriate use of nonpharmacists is essential to adoption and success of diabetes prevention programs.	A traditional chain pharmacy participant explained this decision process as follows: “Our workflow is designed to really generate large volumes of scripts [prescriptions] in a very standard and high-quality, safe way. And so, we do offer pharmacy interventions, and we take our pharmacist out of workflow to have conversations with patients, but . . . we’re really strategic . . . because there’s only so much time that the pharmacist has to have these conversations and conduct these interventions.”

Customer loyalty	A clearly articulated advantage against competition, and alignment of values, is key to a pharmacy’s adoption of diabetes prevention services.	Customer loyalty is a top advantage gained by pharmacy adopters of diabetes prevention programs, but specific characteristics of grocery stores that made delivery of those programs easier was an advantage not seen in other settings.	An independent pharmacy participant said patients feel valued and, “they can leave having learned about diabetes, and physically how to prevent it. And that, ultimately, promotes customer loyalty, which would then promote pharmacy shopping. It’s a domino effect.”

Expanded access and collaboration	Demonstrates and affirms expanded role and value of pharmacies to serve communities. Reaching those without health care access also drives initiation of services.	Pharmacies are focused on expanding health care access to at-risk populations and collaborating with health care teams.	One mass merchant participant said, “I think it’s a great public service, so raising awareness and helping to serve the patient has been really beneficial. I think it helps to engage our pharmacists in a way that they haven’t been engaged previously, and so that professional satisfaction to really have a meaningful clinical conversation with someone who’s unaware that they may be prediabetic.”

### Theme 1: Financial feasibility

Diabetes prevention programs are more likely to be offered at pharmacies if initial financial support is received. Almost half of participants received a grant from a state or local health department or a state pharmacy association to offset initial costs of implementation. Participants who received grant support said funding was critical to mitigate financial risk while launching or expanding their program. Even though no participants were eligible to bill through Medicare, the potential to do so positively influenced the pharmacies’ willingness to offer the program and served as a catalyst for adoption. Most chain pharmacy participants discussed the value of piloting programs with their employees — an opportunity for cost avoidance through self-insured plans and aggregation of data to demonstrate value to payers.

Most adopters of diabetes prevention programs reported no reimbursement beyond grants. Only grocery store participants reported receiving reimbursement for the National DPP lifestyle-change intervention and had better results with payment coverage of diabetes testing than did other interviewees who conducted diabetes prevention programs at other types of pharmacies. Grocery participants secured employer group contracts for diabetes testing (eg, blood glucose test, hemoglobin A_1c_ test) and covered services for their employees through self-insured plans. They also offset testing costs through manufacturer sponsorship and use of clinic staff (eg, dietitians) for medical billing.

Financial sustainability was an important factor many adopters considered when deciding whether to continue services. Participants shared concerns about the inadequacy of the traditional business model, which is built on reimbursement for medications rather than care delivery. Nearly all former adopters or nonadopters expressed willingness to consider offering diabetes prevention programs if a sustainable, scalable model existed.

### Theme 2: Patient participation

Patient participation emerged as a central, unexpected theme. Most participants said buy-in and demand for services were ongoing or anticipated barriers that influenced whether they offered diabetes prevention programs. Their concern for diabetes testing buy-in related to uptake of the service, whereas their concern for the National DPP lifestyle-change intervention centered on enrollment and retention. A recurring concern for patient retention was the 52-week length of National DPP lifestyle-change intervention and related challenges to meet performance measures set by CDC and payers.

Many participants felt that the low level of patient buy-in and demand was fueled by inadequate education and unwillingness to proactively improve one’s health. Other perspectives on why participants may not demand the services included not fully understanding the seriousness of prediabetes, finding time in their schedules to participate, and their social determinants of health. Despite barriers related to patient buy-in and demand, many study participants expressed optimism about identifying solutions. A traditional chain pharmacy nonadopter said, “It’s just figuring out what is really [going to] resonate with the patient, and how do we get them to engage.”

### Theme 3: Operational fit

Participants discussed alignment with existing pharmacy operations as an important factor for adoption. Participants characterized alignment across several areas, including legal, policy, and documentation issues; physical space; standardization and time; staffing; and technology. The most cited legal and policy barrier was the lack of recognition of pharmacists as a provider at the federal level, which affects reimbursement for testing. A few participants cited complicated or nonexistent pharmacy Clinical Laboratory Improvement Amendments (CLIA)-waiver authority in some states, preventing them from offering point-of-care tests (eg, hemoglobin A_1C_). Some discussed complex administrative barriers related to updating pharmacy management systems and executing medical billing.

Participants agreed that clinical and support staff should work to the top of their professional capacity, allowing pharmacists more time for patient care. Most participants carefully considered how and when to use valuable personnel resources, weighing quality, appropriateness, and cost. All grocery store participants employed dietitians, and two-fifths used dietitians as lifestyle coaches for their National DPP lifestyle-change intervention classes. Participants spoke positively about the role of pharmacy interns and residents. Those without access to such personnel noted their value. One independent pharmacy participant said, “We don’t currently have a resident. If I did have a resident, absolutely, that [diabetes prevention program] would be one of the things that I would very adamantly have them participating in.”

Some participants discussed technology. One grocery participant mentioned their company’s proactive effort to develop a “digital and face-to-face option [for the National DPP lifestyle-change intervention], and highly encouraging a combination option. Initial visit in-person and then customize the program based on their needs.”

### Theme 4: Customer loyalty

Many participants stated the primary advantage of offering diabetes prevention programs is increased customer loyalty. Delivery of such services increased trust and good will. Several participants felt delivering these services contributes to positively changing public opinion of pharmacists as care providers.

Several participants saw a clear advantage to offering diabetes prevention programs when doing so clearly aligned with their company’s core values; for nonadopters, making that connection led to a greater consideration of these programs. Grocery store participants discussed their proximity to fresh food and wellness as advantages, beyond using dietitians. One participant said, “because we’re a pharmacy embedded in a supermarket . . . teaching someone how to read labels, teaching someone how to shop correctly, teaching someone how to carb count. I mean, that’s the advantage that we would have.”

### Theme 5: Expanded access and collaboration

Most participants felt pharmacies can positively address prediabetes. Several participants identified disease prevention as critical for their pharmacies, and some mentioned the value of supporting their communities, helping patients — including at-risk and rural communities — to make more informed choices and expand access to enhanced care services. One independent pharmacy participant said, “Anything we can do to help to bring down the rate of diabetes is a great plus, and personally I’m passionate about diabetes prevention . . . [and] want to see chronic disease come down.”

Connection to primary care providers and delivery of team-based care was important, and a few participants mentioned referrals to health departments. One mass merchant participant mentioned a pilot program to conduct diabetes testing for patients and make referrals for enrollment in the National DPP lifestyle-change intervention at local YMCAs. A few participants emphasized the value of team-based care. An independent pharmacy participant said, “The one thing I still hold today is that successful health care for patients doesn’t come from one health care professional; it doesn’t come from the physician; it doesn’t come from the pharmacist; it doesn’t come from the nurse. It comes from working as a team.” Many participants expressed a strong connection to the communities and people they serve; they had a strong desire to “do the right thing.”

## Discussion

Key informant interviews with pharmacy executives identified factors that influence whether pharmacies adopt diabetes prevention programs. Our strongest recommendation to increase pharmacy engagement in diabetes prevention programs is to focus on pharmacies most likely to succeed — such as grocery stores with pharmacies in areas with a high prevalence of diabetes. More work is needed to overcome challenges so more pharmacies can alleviate the public health burden of prediabetes. Literature exists that considers potential solutions to overcome these challenges.

Financial feasibility and sustainable reimbursement are critical for adoption of diabetes prevention programs. Although grant funding is helpful initially, long-term sustainability is needed. One national organization has shifted its grant giving to focus on delivery of services that include community pharmacy (8). The importance of grant funding to pharmacy is not specific to diabetes prevention programs but instead to myriad services such as hepatitis C point-of-care testing and prenatal breastfeeding services (9,10). Previous research explored development of business models for care services delivery in pharmacies (11).

A regional division of a national grocery store chain pharmacy attributed its success in offering diabetes prevention programs in its stores to several factors, including experience in processing third-party payments and support from initial grant funding, which allowed for staff training, participant scholarship opportunities, assistance with medical billing, and access to data management programs (12). CDC has funded all state health departments to advance coverage through Medicaid agencies (12). At least 1 national entity has received CDC funds to implement diabetes prevention in pharmacies (12), but the number of similar grants at the state or local level is unknown. Nine states have full or partial coverage of the National DPP lifestyle-change intervention through Medicaid demonstration projects. Managed care organizations participated in projects that focused on implementation and uptake in 2 states (4). Description of these projects did not specify whether pharmacies were involved.

On November 2, 2017, the Centers for Medicare and Medicaid Services issued the 2018 Physician Fee Schedule final rule, which included a policy that introduced a new benefit to cover the National DPP lifestyle-change intervention for eligible Medicare beneficiaries. The final rule was developed on the basis of a YMCA Diabetes Prevention Program, a pilot study supported by the Center for Medicare and Medicaid Innovation that yielded a cost savings of $2,650 per Medicare enrollee (13). Community pharmacies, among others, can apply to become “suppliers,” which can yield reimbursement from $195 to $670 per enrolled beneficiary, depending on achievement of performance goal (14). However, Medicare does not cover pharmacist-conducted diabetes testing to determine eligibility for enrollment in the National DPP lifestyle-change intervention, even though half of participants enrolled by 1 supplier are required to obtain a diabetes blood test for enrollment rather than a paper-based assessment. To improve pharmacy participation, Medicare should use their existing authority to allow pharmacies to supply patient care services with reimbursement for diabetes testing, among other care services. Modernization of Medicare regulations to ensure pharmacies and pharmacists are covered to deliver care services is an important policy change consistent with a recent executive order focused on improving Medicare (15).

As of October 2018, CDC showed that fewer than 250,000 people had enrolled in the National DPP lifestyle-change intervention since the program began in 2010. This number represents less than 1% of the nation’s population with prediabetes. The problem of scaling diabetes prevention programs to those eligible is a broad public health challenge, not one specific to pharmacy. The National DPP lifestyle-change intervention was piloted at 5 New York City recreation sites that served men from disadvantaged neighborhoods. Extensive recruitment efforts were conducted with the guidance of an advisory panel and incentives for participation, yet recruitment was still challenging (16). One example of successful recruitment took place at an African American church. This church recruited participants by adapting the National DPP lifestyle-change intervention to include faith-based references for those who were eligible to participate (17). In another intervention, health promotion led by barbers in coordination with medication management by pharmacists in barbershops led to reductions in uncontrolled hypertension (18). Pharmacy could use its previous successes in addressing hypertension and other health conditions by offering accessible care services for patients and applying such knowledge to diabetes prevention.

A 2013 study of the YMCA and UnitedHealth Group’s National DPP lifestyle-change intervention, in collaboration with CDC, indicated that patient engagement was its greatest challenge (19). The greatest success for enrollment in this study came from community- and employer-based diabetes testing events coupled with onsite counseling and enrollment to leverage “teachable moments” (19). A 2019 review of findings from 6 CDC-funded national organizations demonstrated that encouraging self-referral or word of mouth as a recruitment strategy, providing nonmonetary incentives to participants, and using cultural adaptations to address participants’ needs were significantly associated with higher levels of attendance for those participating in the National DPP lifestyle-change intervention (20). Veterans who participated in an intensive, multifaceted online version of the National DPP lifestyle-change intervention were more likely to complete 8 or more sessions than those participating in person (87% online vs 59% in person); however both groups had similar weight loss (21). Among low-income populations, a digital, modified 52-week offering of the National DPP lifestyle-change intervention resulted in weight loss (22). Pharmacies and other health care providers should consider digital delivery of the National DPP lifestyle-change intervention, and payers, including Medicare, should broaden their coverage requirements accordingly.

Operational fit is important for patient care services delivered in pharmacies, not specific to diabetes prevention (23). For instance, vaccination has successfully been integrated into pharmacies, with most pharmacies offering walk-in services, while maximizing staff, physical space, and resources to meet patient needs (24). Researchers attribute the success of pharmacist-delivered vaccination to 5 fundamental factors, including 2 factors that are particularly relevant for this study: a policy/legal platform and a sustainable business model (25). In the United Kingdom, researchers identified barriers similar to those in our study, including the pressure to find time for pharmacy staff to deliver diabetes prevention services, space challenges, and lack of access to medical records (26). An article describing the experience of delivering a diabetes prevention program at a regional division of a national grocery store chain pharmacy highlighted the importance of using the entire pharmacy workforce for program sustainability (12).

The relationship between pharmacists and patients in the loyalty-building path has shown that trust in pharmacists is the most important driver of satisfaction and store loyalty. Experts largely agree that loyalty is made up of both attitudinal (eg, intentional, cognitive) factors and behavioral (eg, purchasing) factors (27).

Regardless of whether customers engage in diabetes prevention, the existence of diabetes prevention programs at pharmacies may increase customer loyalty and appreciation for the pharmacy brand. In a study that defined loyal patients as those who filled all their prescription drugs at a single pharmacy during the first year of diabetes treatment, the loyal patients were more adherent to their medication use regimen than patients who used multiple pharmacies (28). In Canada, participation in pharmacy-based inducement programs (eg, cash, coupons, discounts, gifts, or points as incentives) among new statin users was associated with better medication adherence than among a control group with no inducement programs (29). In a cohort study that used data from a health insurance board in Canada, pharmacy loyalty may have been associated with improved adherence to antipsychotic medication and treatment implementation among people with severe mental illness (30). These studies indicate that not only is the relationship between pharmacists and patients important but also that efforts made by pharmacies to improve customer loyalty can improve health outcomes.

A study describing a diabetes prevention program at a grocery store with a pharmacy credited the success of the program in part to the uniqueness of practice locations, proximity to food, and convenience (12). Furthermore, the study suggested that pharmacists may have greater access to populations that need to increase their awareness of diabetes prevention than other nonpharmacy care providers. 

Collaboration among pharmacists and others is critical to improve chronic disease outcomes, and pharmacists should be included in value-driven, collaborative models such as patient-centered medical homes and accountable care organizations. Furthermore, fostering strong relationships between physicians and pharmacists on care teams is well documented as critical to patient care (31). Specific to diabetes prevention, Medicare beneficiary enrollment in a YMCA National DPP lifestyle-change intervention was improved when a physician made a point-of-care referral rather than retrospective methods (eg, use of electronic medical record systems to identify eligible patients via a registry) (32).

In the United Kingdom study, stakeholders (eg, community pharmacists, general practitioners, and commissioners) viewed pharmacies as a place for prescription services and not necessarily as a place for diabetes management. However, those stakeholders agreed that pharmacies provided patients with increased choices for their primary care services and have potential for greater reach to certain populations given the normalized, nonjudgmental setting of a pharmacy. Additionally, the stakeholders felt that community pharmacies could be locations for individualizing interventions as an alternative to the traditional group in-person format of the National DPP (26). Such findings are consistent with the perception among some people that community pharmacies and retail clinics are more accessible and less stigmatizing than traditional sites for delivery of HIV testing (33).

Our study gave equal weight to the perspectives of adopters, nonadopters, and former adopters, and themes were weighted equally. However, we were unable to identify nonadopters and former adopters among grocery store participants (they were all adopters in our study) and interviewed fewer traditional chain pharmacy and mass merchant participants than participants from other pharmacy types. We attempted to include those who adopted the National DPP lifestyle-change intervention and adopters of other lifestyle change programs, but only 1 adopter in the study delivered a lifestyle-change intervention other than the National DPP, and it was a modified, shortened version based on the National DPP curriculum. The views of the participants may not entirely reflect the views of all decision makers in their company or the final decision makers in their company. However, the included participants represented approximately half of pharmacies in the United States in 2018. Finally, this study did not garner input from patients of the National DPP lifestyle-change intervention, even though a major theme emerged related to patient buy-in. To that end, this study did not closely examine provider referrals or their effect on patient perception of pharmacy services. Further research is warranted in this area.

Addressing the threat of diabetes is a national public health concern. Reducing or eliminating barriers that prevent pharmacies from fully adopting diabetes testing programs and the National DPP lifestyle-change intervention could have a profound effect on patient care. Our study identified factors that influence pharmacy decision-maker adoption of diabetes testing programs and the National DPP lifestyle-change intervention. Pharmacy decision makers have many competing interests in patient care programs. Diabetes testing and the National DPP lifestyle-change intervention are not of interest to all pharmacies; however, our study indicates that several companies are offering such services and want to expand. Pharmacies are a strong asset in public health’s efforts to address diabetes prevention nationally; however, given the current legal, regulatory, and financial landscape, some pharmacies may not see the value in participation without removal of these barriers.
